# Ammonium iron(III) phosphate(V) fluoride, (NH_4_)_0.5_[(NH_4_)_0.375_K_0.125_]FePO_4_F, with ammonium partially substituted by potassium

**DOI:** 10.1107/S1600536808042499

**Published:** 2008-12-20

**Authors:** Lei Wang, Yan Zhou, Ya-Xi Huang, Jin-Xiao Mi

**Affiliations:** a Department of Materials Science and Engineering, College of Materials, Xiamen University, Xiamen 361005, Fujian Province, People’s Republic of China

## Abstract

The title compound, ammonium potassium iron(III) phosphate fluoride, (NH_4_)_0.875_K_0.125_FePO_4_F, is built from zigzag chains _∞_^1^{[FeO_4_F_2_]^7−^}, with Fe^3+^ in a distorted octahedral coordination, extending along both the [011] and [0
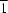
1] directions. These chains are made up of alternating *trans*-[FeO_4_F_2_] and *cis*-[FeO_4_F_2_] octa­hedra *via* shared F-atom corners, and are linked by PO_4_ tetra­hedra, resulting in an open-framework structure with channels along the [010] and [100] directions. There are two crystallographically independent ammonium sites: one in the [010] channels and the other, partially substituted by K^+^ ions, in the [100] channels. The ammonium in the [010] channels is fixed to the framework *via* eight hydrogen bonds (six N—H⋯O and two N—H⋯F).

## Related literature

For general background, see: Hagerman & Poeppelmeier (1995 ▶). For related structures, see: Loiseau *et al.* (1994 ▶) for (NH_4_)Fe(PO_4_)F; Loiseau *et al.* (2000 ▶) for (NH_4_)Ga(PO_4_)F; Alda *et al.* (2003 ▶) for (NH_4_)V(PO_4_)F; Slovokhotova *et al.* (1991 ▶) for KAl(PO_4_)F; Harrison *et al.* (1995 ▶) for KGa(PO_4_)F; Matvienko *et al.* (1979 ▶) for KFe(PO_4_)F; Slobodyanik *et al.* (1991 ▶) for KCr(PO_4_)F; Tordjman *et al.* (1974 ▶) for K(TiO)(PO_4_).

## Experimental

### 

#### Crystal data

(NH_4_)_0.5_[(NH_4_)_0.375_K_0.125_]FePO_4_F
*M* *_r_* = 190.49
Orthorhombic, 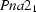

*a* = 12.9402 (4) Å
*b* = 6.4382 (2) Å
*c* = 10.6428 (3) Å
*V* = 886.67 (5) Å^3^
*Z* = 8
Mo *K*α radiation
μ = 3.82 mm^−1^
*T* = 173 (2) K
0.12 × 0.09 × 0.09 mm

#### Data collection

Oxford Diffraction CCD area-detector diffractometer
Absorption correction: numerical (**CrysAlis RED**; Oxford Diffraction, 2005 ▶) *T*_min_ = 0.657, *T*_max_ = 0.725
5371 measured reflections
2162 independent reflections
1636 reflections with *I* > 2σ(*I*)
*R*_int_ = 0.040

#### Refinement

*R*[*F* ^2^ > 2σ(*F* ^2^)] = 0.031
*wR*(*F* ^2^) = 0.054
*S* = 0.88
2162 reflections
155 parameters
5 restraints
H-atom parameters constrained
Δρ_max_ = 0.52 e Å^−3^
Δρ_min_ = −0.56 e Å^−3^

### 

Data collection: *CrysAlis CCD* (Oxford Diffraction, 2005 ▶); cell refinement: *CrysAlis CCD*; data reduction: *CrysAlis RED* (Oxford Diffraction, 2005 ▶); program(s) used to solve structure: *SHELXS97* (Sheldrick, 2008 ▶); program(s) used to refine structure: *SHELXL97* (Sheldrick, 2008 ▶); molecular graphics: *DIAMOND* (Brandenburg, 2005 ▶) and *ATOMS* (Dowty, 2004 ▶); software used to prepare material for publication: *SHELXL97*.

## Supplementary Material

Crystal structure: contains datablocks I, global. DOI: 10.1107/S1600536808042499/fi2066sup1.cif

Structure factors: contains datablocks I. DOI: 10.1107/S1600536808042499/fi2066Isup2.hkl

Additional supplementary materials:  crystallographic information; 3D view; checkCIF report

## Figures and Tables

**Table 1:** Selected geometric parameters (Å, °)

Fe1—O5^i^	1.893 (4)
Fe1—O6	1.955 (4)
Fe1—O8^ii^	1.963 (4)
Fe1—F1^i^	1.988 (4)
Fe1—F2	2.010 (4)
Fe1—O7	2.088 (4)
Fe2—F2	1.936 (4)
Fe2—O3	1.944 (5)
Fe2—O1	1.986 (5)
Fe2—F1	2.001 (4)
Fe2—O2	2.003 (4)
Fe2—O4	2.055 (3)
P1—O1^i^	1.531 (5)
P1—O7	1.532 (4)
P1—O6^iii^	1.538 (4)
P1—O3^iii^	1.551 (5)
P2—O2	1.530 (4)
P2—O8	1.533 (5)
P2—O4^iv^	1.539 (4)
P2—O5	1.547 (5)

Symmetry codes: (i) 
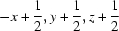
; (ii) 
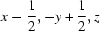
; (iii) 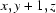; (iv) 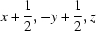.

**Table 2:** Hydrogen-bond geometry (Å, °)

*D*—H⋯*A*	*D*—H	H⋯*A*	*D*⋯*A*	*D*—H⋯*A*
N2—H1⋯F1^i^	0.86 (4)	2.37 (5)	3.079 (6)	140 (4)
N2—H1⋯O4^i^	0.86 (4)	2.39 (3)	3.124 (6)	144 (4)
N2—H1⋯O1^i^	0.86 (4)	2.54 (5)	3.159 (7)	130 (4)
N2—H2⋯O2	0.87 (4)	2.13 (4)	2.956 (6)	159 (4)
N2—H2⋯F2	0.87 (4)	2.22 (5)	2.744 (6)	118 (4)
N2—H3⋯O8^iii^	0.85 (4)	2.29 (5)	2.775 (6)	117 (4)
N2—H3⋯O3^iii^	0.85 (4)	2.39 (5)	3.088 (7)	139 (4)
N2—H4⋯O7^vii^	0.84 (3)	2.06 (3)	2.823 (5)	151 (4)

Symmetry codes: (i) 
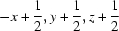
; (iii) 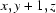; (vii) 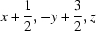.

**Fig. 1.**
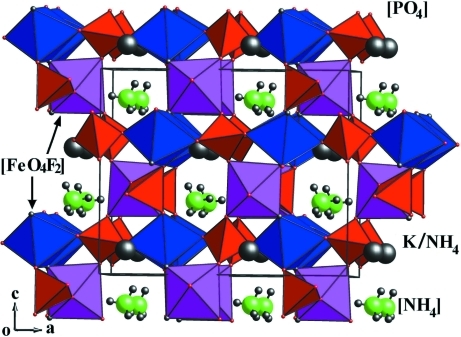
Unit cell of (NH4)((NH4)0.75K0.25) [Fe2(PO4)2F2] in a view along b. Trans-[FeO4F2], in blue octahedra; cis-[FeO4F2], in pink octahedra; PO4, in red tetrahedra; black spheres: disordered K/NH4; green spheres: NH4.

**Fig. 2.**
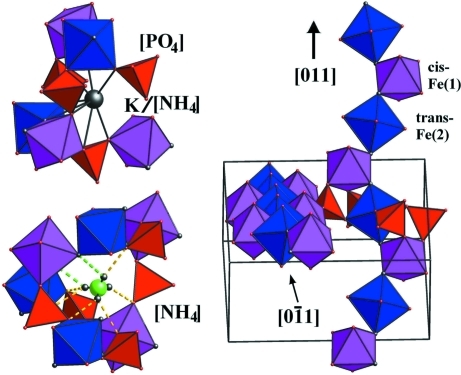
Left: (1). Coordination environment of eight coordinated potassium/ammonium ions(upper); (2). Coordination environment of ammonium ions via hydrogen bonds(down).Right: chains built up of alternate trans-[FeO4F2] and cis-[FeO4F2] octahedra running along [011] and [0-11] respectively;

**Fig. 3.**
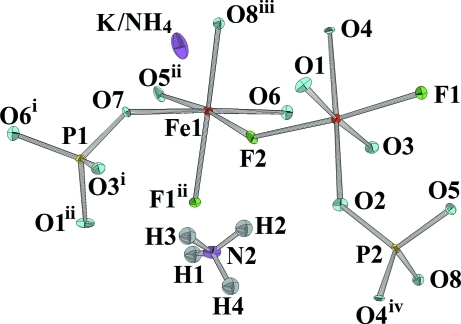
Coordination environment of Fe and P atoms, with displacement ellipsoids drawn at the 50% probability level (symmetry codes: (i) x, 1+y, z; (ii) 1/2-x, 1/2+y, 1/2+z; (iii) -1/2+x, 1/2-y, z; (iv) 1/2+x, 1/2-y, z).

## supplementary crystallographic information

### Comment

Compounds of the KTiOPO_4_ (KTP) group have attracted great attention in the past decades owing to their non-linear optical properties, and became a large family of compositions with formula MM'OXO_4_ (M = Na, K, Rb, Cs or Tl; M' = Ti, Ge, Sn and X = P or As), where M and M' can be partially replaced by two or more different cations (Hagerman *et al.*, 1995). Tetravalent M' ions can be replaced by trivalent ions such as Fe^3+^ and Cr^3+^ with oxygen being substituted simultaneously by fluorine for charge balance. Many examples of fluorophosphates with the KTP-type structure are known, such as (NH_4_)Fe(PO_4_)F, (NH_4_)Ga(PO_4_)F (Loiseau *et al.*, 1994, 2000 ), and (NH_4_)V(PO_4_)F (Alda *et al.*, 2003) in the ammonium series, and KAl(PO_4_)F (Slovokhotova *et al.*, 1991), KGa(PO_4_)F (Harrison *et al.*, 1995), KFe(PO_4_)F (Matvienko *et al.*, 1979), and KCr(PO_4_)F (Slobodyanik *et al.*, 1991) in the potassium series. However, to the best of our knowledge, sodium fluorophosphate compounds with the KTP-type structure have not been reported to date. Our attempt to synthesize KTP-type sodium fluorophophates failed as well, instead we obtained a solid-solution ammonium fluorophosphate, (NH_4_)((NH_4_)_0.75_K_0.25_)[Fe_2_(PO_4_)_2_F_2_], partially substituted by potassium.

The crystal structure of the title compound is built from one-dimensional zigzag ferric octahedral chains _∞_^1^{[FeO_4_F_2_]^7-^} (see Fig. 1) linked by PO_4_ tetrahedra via common O-corners, resulting in a three dimensional open framework structure (see Fig.2). The zigzag ferric octahedral chains are made up of alternating *trans*-[FeO_4_F_2_] and *cis*-[FeO_4_F_2_] octahedra via common fluorine-corners, and are along both [011] and [0-11] directions. Each PO_4_ tetrahedron shares all oxygen corners with four neighboring [FeO_4_F_2_] octahedra, and each [FeO_4_F_2_] octahedron shares its four oxygen atoms with four PO_4_ tetrahedra. The negative charge of the framework is compensated by ammonium ions and potassium cations. Two crystallographically independent ammonium sites are found. One resides in the channels along [010], linking to the framework via hydrogen bonds (N-H···O(F)). If this ammonium ion is considered as a normal cation, its coordination number is 8 with an average bond distance of 2.969Å (i.e., to neighboring oxygen and fluorine atoms). The other ammonium site, partially occupied by K^+^ ions, resides in the channels along [100]. The potassium (ammonium) has a coordination number of 8 with a mean bond distance of 2.882Å. Therefore, the ammonium site in the [010] channels is slightly larger in size than its counterpart in the [100] channels. Consequently, potassium ions preferentially substitute for the ammonium ions in the [100] channels. We suggest that the potassium-to-ammonium ratio in the title compound is controlled by the size effect. It is possible that the size effect in hydrothermally synthesized crystals at lower temperatures is more pronounced than those from a solid state reaction route.

### Experimental

Transparent, colorless single crystals of the title compound were synthesized hydrothermally. A mixture of 0.055 g NaBF_4_, 1.035 g NH_4_H_2_PO_4_, 0.342 g NH_4_HF_2_ and 0.048 g Fe_2_O_3_ in an approximate molar ratio of Na: NH_4_: P: Fe = 0.5: 15: 9: 6, was dissolved in 9 ml distilled water while stirring. The prepared solution was transferred to a Teflon-lined stainless steel autoclave (internal volume 30 ml, degree of filling 33%) and held at 453 K for three days under autogenous pressure. Then the autoclave was cooled to room temperature by turning off the power. Products were filtered off, washed with distilled water and dried at room temperature. The presence of potassium in the crystal that was used for single crystal X-ray data collection, was determined by semi-quantitative chemical analysis on an Oxford Instruments Energy Dispersive Spectrometer(EDS)(calcd K: 2.56 %, Obsd K: ~3%). Sodium was not detectable in the crystal by EDS. It is supposed that potassium should be introduced into the sample as impurities in the reagents.

### Refinement

There are two possible space groups Pna2_1_(abc) and Pnna(acb) for the KTP-type compounds. Initially, the centrosymmetric Pnna( No.52) space group was selected according to the observed systematic absences. All the framework atoms fit to the centrosymmetric model, except that the ammonium ions resided in the channel are in disordered manner and have abnormally short (1.46 Å) N–N distances. Furthermore, the structural refinement converged to only R1(gt)=0.0783 and *wR*(all)=0.1813, which are much higher than those from the noncentrosymmetric model (i.e., all anisotropic atoms resulting in R1(gt)=0.031,and *wR*(all)=0.054 using 155 parameters). Therefore, the noncentrosymmetric space group Pna2_1_(No.33) was chosen to solve and refine the crystal structure as an inversion twin with twin components 0.48 (3)/0.52 (3). During the structure refinement, five constrained parameters were set, one for the constraint of N1 and K1 atoms (i.e., EXYZ and EADP) sharing the same site, the other four for geometrical constraints (i.e., HFIX: fixed bond distance 0.89 Å) of N2–H1, N2–H2, N2–H3, and N2–H4, respectively. Hydrogen coordinate parameters linked to N2 were obtained from difference electron density synthesis and refined by constrained N–H bond distances, their displacement parameters refined via setting to a common variable. Hydrogen atoms linked to N1 were not determined in the present paper.

### Crystal data

**Table:**

(NH_4_)_0.875_K_0.125_FePO_4_F	*F*(000) = 752
*M**_r_* = 190.49	*D*_x_ = 2.854 Mg m^−^^3^
Orthorhombic, *P**n**a*2_1_	Mo *K*α radiation, λ = 0.71073 Å
Hall symbol: P 2c -2n	Cell parameters from 5001 reflections
*a* = 12.9402 (4) Å	θ = 3.0–32.5°
*b* = 6.4382 (2) Å	µ = 3.82 mm^−^^1^
*c* = 10.6428 (3) Å	*T* = 173 K
*V* = 886.67 (5) Å^3^	Prism, colorless
*Z* = 8	0.12 × 0.09 × 0.09 mm

### Data collection

**Table:**

Oxford Diffraction CCD area-detector diffractometer	2162 independent reflections
Radiation source: fine-focus sealed tube	1636 reflections with *I* > 2σ(*I*)
graphite	*R*_int_ = 0.040
326 images,Δω=1°, Exp time: 40 s. scans	θ_max_ = 32.5°, θ_min_ = 2.5°
Absorption correction: numerical (*CrysAlis RED*; Oxford Diffraction, 2005)	*h* = −19→16
*T*_min_ = 0.657, *T*_max_ = 0.725	*k* = −4→9
5371 measured reflections	*l* = −10→16

### Refinement

**Table:**

Refinement on *F*^2^	Primary atom site location: structure-invariant direct methods
Least-squares matrix: full	Secondary atom site location: difference Fourier map
*R*[*F*^2^ > 2σ(*F*^2^)] = 0.031	Hydrogen site location: inferred from neighbouring sites
*wR*(*F*^2^) = 0.054	H-atom parameters constrained
*S* = 0.88	*w* = 1/[σ^2^(*F*_o_^2^) + (0.0164*P*)^2^] where *P* = (*F*_o_^2^ + 2*F*_c_^2^)/3
2162 reflections	(Δ/σ)_max_ < 0.001
155 parameters	Δρ_max_ = 0.52 e Å^−^^3^
5 restraints	Δρ_min_ = −0.56 e Å^−^^3^

### Special details

**Table:**

Geometry. All esds (except the esd in the dihedral angle between two l.s. planes) are estimated using the full covariance matrix. The cell esds are taken into account individually in the estimation of esds in distances, angles and torsion angles; correlations between esds in cell parameters are only used when they are defined by crystal symmetry. An approximate (isotropic) treatment of cell esds is used for estimating esds involving l.s. planes.
Refinement. Refinement of F^2^ against ALL reflections. The weighted R-factor wR and goodness of fit S are based on F^2^, conventional R-factors R are based on F, with F set to zero for negative F^2^. The threshold expression of F^2^ > 2sigma(F^2^) is used only for calculating R-factors(gt) etc. and is not relevant to the choice of reflections for refinement. R-factors based on F^2^ are statistically about twice as large as those based on F, and R- factors based on ALL data will be even larger. The crystal structure of the title compound was refined by a inversion twin matrix so the Flack parameter is equal to zero.

### Fractional atomic coordinates and isotropic or equivalent isotropic displacement parameters (Å^2^)

**Table:**

	*x*	*y*	*z*	*U*_iso_*/*U*_eq_	Occ. (<1)
Fe1	0.11299 (4)	0.48776 (11)	0.41908 (14)	0.00427 (10)	
Fe2	0.25117 (7)	0.25076 (13)	0.16711 (14)	0.00434 (9)	
K1	0.1161 (2)	0.7780 (4)	0.1155 (3)	0.0243 (9)	0.249 (5)
N1	0.1161 (2)	0.7780 (4)	0.1155 (3)	0.0243 (9)	0.75
N2	0.4008 (3)	0.6749 (6)	0.3543 (4)	0.0094 (7)	
P1	0.18531 (6)	0.9964 (2)	0.4152 (2)	0.00453 (15)	
P2	0.49699 (14)	0.17105 (12)	0.1701 (2)	0.00426 (15)	
F1	0.2694 (3)	0.0303 (7)	0.0365 (3)	0.0067 (8)	
F2	0.2233 (3)	0.4707 (7)	0.2864 (3)	0.0070 (8)	
O1	0.2426 (3)	0.4522 (9)	0.0261 (4)	0.0082 (9)	
O2	0.4023 (3)	0.3113 (6)	0.1847 (4)	0.0075 (8)	
O3	0.2540 (3)	0.0417 (9)	0.2989 (4)	0.0076 (9)	
O4	0.0953 (3)	0.1996 (6)	0.1474 (4)	0.0056 (8)	
O5	0.4803 (4)	0.0316 (8)	0.0535 (3)	0.0095 (9)	
O6	0.1173 (3)	0.1868 (5)	0.4430 (4)	0.0069 (8)	
O7	0.1172 (3)	0.8075 (5)	0.3867 (4)	0.0061 (8)	
O8	0.5104 (3)	0.0264 (8)	0.2834 (3)	0.0057 (9)*	
H1	0.372 (3)	0.678 (8)	0.427 (3)	0.020 (7)*	
H2	0.386 (4)	0.580 (7)	0.299 (4)	0.020 (7)*	
H3	0.385 (4)	0.780 (6)	0.310 (4)	0.020 (7)*	
H4	0.462 (2)	0.634 (8)	0.365 (5)	0.020 (7)*	

### Atomic displacement parameters (Å^2^)

**Table:**

	*U*^11^	*U*^22^	*U*^33^	*U*^12^	*U*^13^	*U*^23^
Fe1	0.0045 (2)	0.0046 (2)	0.00376 (18)	0.0002 (3)	−0.0004 (5)	0.0001 (2)
Fe2	0.00452 (18)	0.00472 (17)	0.00377 (17)	0.00037 (17)	0.00040 (18)	0.0003 (2)
K1	0.0311 (16)	0.0146 (14)	0.0271 (14)	−0.0069 (11)	0.0077 (11)	−0.0039 (10)
N1	0.0311 (16)	0.0146 (14)	0.0271 (14)	−0.0069 (11)	0.0077 (11)	−0.0039 (10)
N2	0.0038 (17)	0.0161 (18)	0.0083 (17)	−0.0023 (15)	0.0024 (14)	0.0011 (15)
P1	0.0048 (4)	0.0048 (3)	0.0039 (4)	−0.0008 (6)	−0.0017 (9)	0.0010 (3)
P2	0.0041 (4)	0.0046 (3)	0.0041 (3)	−0.0001 (6)	−0.0001 (3)	−0.0030 (7)
F1	0.0066 (18)	0.0067 (14)	0.0070 (16)	−0.0007 (14)	0.0021 (13)	−0.0002 (13)
F2	0.0077 (18)	0.0096 (15)	0.0037 (15)	−0.0016 (15)	0.0019 (13)	−0.0036 (13)
F1	0.0066 (18)	0.0067 (14)	0.0070 (16)	−0.0007 (14)	0.0021 (13)	−0.0002 (13)
F2	0.0077 (18)	0.0096 (15)	0.0037 (15)	−0.0016 (15)	0.0019 (13)	−0.0036 (13)
O1	0.008 (2)	0.0123 (18)	0.0045 (19)	0.0027 (19)	0.0029 (15)	0.0042 (17)
O2	0.005 (2)	0.0119 (15)	0.005 (2)	−0.0012 (15)	0.0027 (18)	0.0014 (15)
O3	0.007 (2)	0.0091 (18)	0.0067 (19)	0.0002 (18)	0.0000 (14)	0.0012 (17)
O4	0.0036 (19)	0.0049 (14)	0.008 (2)	0.0006 (13)	−0.0034 (17)	0.0000 (14)
O5	0.0102 (18)	0.0095 (15)	0.0089 (16)	0.0032 (14)	−0.0035 (12)	−0.0052 (11)
O6	0.0095 (18)	0.0063 (13)	0.005 (2)	0.0009 (17)	0.0009 (19)	0.0017 (13)
O7	0.0070 (17)	0.0053 (13)	0.006 (2)	0.0005 (16)	−0.0013 (19)	−0.0034 (12)

### Geometric parameters (Å, °)

**Table:**

Fe1—O5^i^	1.893 (4)	N2—O7^iv^	2.823 (5)
Fe1—O6	1.955 (4)	N2—O2	2.956 (6)
Fe1—O8^ii^	1.963 (4)	N2—F1^i^	3.079 (6)
Fe1—F1^i^	1.988 (4)	N2—O3^iii^	3.088 (7)
Fe1—F2	2.010 (4)	N2—O4^i^	3.124 (6)
Fe1—O7	2.088 (4)	N2—O1^i^	3.159 (7)
Fe2—F2	1.936 (4)	P1—O1^i^	1.531 (5)
Fe2—O3	1.944 (5)	P1—O7	1.532 (4)
Fe2—O1	1.986 (5)	P1—O6^iii^	1.538 (4)
Fe2—F1	2.001 (4)	P1—O3^iii^	1.551 (5)
Fe2—O2	2.003 (4)	P2—O2	1.530 (4)
Fe2—O4	2.055 (3)	P2—O8	1.533 (5)
K1—F1^iii^	2.697 (5)	P2—O4^v^	1.539 (4)
K1—O5^ii^	2.739 (6)	P2—O5	1.547 (5)
K1—O4^iii^	2.749 (4)	F1—Fe1^vi^	1.988 (4)
K1—O1	2.825 (6)	F1—K1^vii^	2.697 (5)
K1—O7	2.893 (5)	O1—P1^vi^	1.531 (5)
K1—O8^ii^	2.985 (5)	O3—P1^vii^	1.551 (5)
K1—F2	3.025 (5)	O3—K1^vii^	3.142 (6)
K1—O3^iii^	3.142 (6)	O4—P2^ii^	1.539 (4)
N2—H1	0.86 (2)	O4—K1^vii^	2.749 (4)
N2—H2	0.87 (2)	O5—Fe1^vi^	1.893 (4)
N2—H3	0.85 (2)	O5—K1^v^	2.739 (6)
N2—H4	0.84 (2)	O6—P1^vii^	1.538 (4)
N2—F2	2.744 (6)	O8—Fe1^v^	1.963 (4)
N2—O8^iii^	2.775 (6)	O8—K1^v^	2.985 (5)
			
O5^i^—Fe1—O6	93.9 (2)	F1^iii^—K1—O3^iii^	56.70 (12)
O5^i^—Fe1—O8^ii^	97.58 (10)	O5^ii^—K1—O3^iii^	155.13 (16)
O6—Fe1—O8^ii^	94.0 (2)	O4^iii^—K1—O3^iii^	56.31 (13)
O5^i^—Fe1—F1^i^	89.56 (19)	O1—K1—O3^iii^	106.34 (16)
O6—Fe1—F1^i^	91.87 (18)	O7—K1—O3^iii^	48.85 (13)
O8^ii^—Fe1—F1^i^	170.45 (17)	O8^ii^—K1—O3^iii^	104.06 (15)
O5^i^—Fe1—F2	172.51 (19)	F2—K1—O3^iii^	73.71 (16)
O6—Fe1—F2	90.97 (18)	H1—N2—H2	122 (5)
O8^ii^—Fe1—F2	87.76 (17)	H1—N2—H3	112 (5)
F1^i^—Fe1—F2	84.57 (10)	H2—N2—H3	98 (5)
O5^i^—Fe1—O7	89.7 (2)	H1—N2—H4	107 (5)
O6—Fe1—O7	176.27 (15)	H2—N2—H4	95 (5)
O8^ii^—Fe1—O7	86.68 (19)	H3—N2—H4	124 (5)
F1^i^—Fe1—O7	87.03 (17)	O1^i^—P1—O7	110.8 (3)
F2—Fe1—O7	85.37 (17)	O1^i^—P1—O6^iii^	110.4 (3)
F2—Fe2—O3	92.1 (2)	O7—P1—O6^iii^	109.98 (13)
F2—Fe2—O1	90.43 (16)	O1^i^—P1—O3^iii^	107.56 (14)
O3—Fe2—O1	176.3 (3)	O7—P1—O3^iii^	108.7 (3)
F2—Fe2—F1	175.3 (2)	O6^iii^—P1—O3^iii^	109.4 (3)
O3—Fe2—F1	90.44 (16)	O2—P2—O8	111.7 (3)
O1—Fe2—F1	86.9 (2)	O2—P2—O4^v^	111.00 (12)
F2—Fe2—O2	88.75 (18)	O8—P2—O4^v^	111.0 (2)
O3—Fe2—O2	92.82 (18)	O2—P2—O5	108.2 (3)
O1—Fe2—O2	89.87 (19)	O8—P2—O5	107.11 (13)
F1—Fe2—O2	95.06 (17)	O4^v^—P2—O5	107.7 (3)
F2—Fe2—O4	90.05 (17)	Fe1^vi^—F1—Fe2	128.6 (2)
O3—Fe2—O4	88.90 (18)	Fe1^vi^—F1—K1^vii^	132.5 (2)
O1—Fe2—O4	88.46 (18)	Fe2—F1—K1^vii^	97.14 (16)
F1—Fe2—O4	86.05 (18)	Fe2—F2—Fe1	129.3 (2)
O2—Fe2—O4	177.9 (2)	Fe2—F2—K1	99.74 (15)
F1^iii^—K1—O5^ii^	146.65 (16)	Fe1—F2—K1	93.52 (16)
F1^iii^—K1—O4^iii^	61.07 (13)	P1^vi^—O1—Fe2	132.1 (3)
O5^ii^—K1—O4^iii^	133.26 (16)	P1^vi^—O1—K1	118.3 (3)
F1^iii^—K1—O1	85.20 (17)	Fe2—O1—K1	105.24 (19)
O5^ii^—K1—O1	75.57 (15)	P2—O2—Fe2	131.1 (2)
O4^iii^—K1—O1	146.27 (16)	P1^vii^—O3—Fe2	134.0 (3)
F1^iii^—K1—O7	105.53 (14)	P1^vii^—O3—K1^vii^	94.0 (2)
O5^ii^—K1—O7	106.92 (14)	Fe2—O3—K1^vii^	85.15 (16)
O4^iii^—K1—O7	79.17 (13)	P2^ii^—O4—Fe2	135.1 (2)
O1—K1—O7	112.44 (15)	P2^ii^—O4—K1^vii^	129.4 (2)
F1^iii^—K1—O8^ii^	156.77 (16)	Fe2—O4—K1^vii^	94.26 (14)
O5^ii^—K1—O8^ii^	51.09 (8)	P2—O5—Fe1^vi^	141.5 (3)
O4^iii^—K1—O8^ii^	121.95 (15)	P2—O5—K1^v^	98.0 (2)
O1—K1—O8^ii^	88.83 (16)	Fe1^vi^—O5—K1^v^	118.9 (2)
O7—K1—O8^ii^	56.47 (12)	P1^vii^—O6—Fe1	141.4 (3)
F1^iii^—K1—F2	104.14 (16)	P1—O7—Fe1	139.9 (2)
O5^ii^—K1—F2	87.88 (16)	P1—O7—K1	104.6 (2)
O4^iii^—K1—F2	128.06 (15)	Fe1—O7—K1	95.74 (14)
O1—K1—F2	56.72 (12)	P2—O8—Fe1^v^	133.1 (3)
O7—K1—F2	55.97 (11)	P2—O8—K1^v^	88.85 (19)
O8^ii^—K1—F2	54.57 (13)	Fe1^v^—O8—K1^v^	95.74 (18)

Symmetry codes: (i) −*x*+1/2, *y*+1/2, *z*+1/2; (ii) *x*−1/2, −*y*+1/2, *z*; (iii) *x*, *y*+1, *z*; (iv) *x*+1/2, −*y*+3/2, *z*; (v) *x*+1/2, −*y*+1/2, *z*; (vi) −*x*+1/2, *y*−1/2, *z*−1/2; (vii) *x*, *y*−1, *z*.

### Hydrogen-bond geometry (Å, °)

**Table:**

*D*—H···*A*	*D*—H	H···*A*	*D*···*A*	*D*—H···*A*
N2—H1···F1^i^	0.86 (4)	2.37 (5)	3.079 (6)	140 (4)
N2—H1···O4^i^	0.86 (4)	2.39 (3)	3.124 (6)	144 (4)
N2—H1···O1^i^	0.86 (4)	2.54 (5)	3.159 (7)	130 (4)
N2—H2···O2	0.87 (4)	2.13 (4)	2.956 (6)	159 (4)
N2—H2···F2	0.87 (4)	2.22 (5)	2.744 (6)	118 (4)
N2—H3···O8^iii^	0.85 (4)	2.29 (5)	2.775 (6)	117 (4)
N2—H3···O3^iii^	0.85 (4)	2.39 (5)	3.088 (7)	139 (4)
N2—H4···O7^iv^	0.84 (3)	2.06 (3)	2.823 (5)	151 (4)

Symmetry codes: (i) −*x*+1/2, *y*+1/2, *z*+1/2; (iii) *x*, *y*+1, *z*; (iv) *x*+1/2, −*y*+3/2, *z*.
